# Integrated Absorption Spectroscopic Measurement of 2-Nitrophenol and Naphthalene

**DOI:** 10.3390/ijms26209904

**Published:** 2025-10-11

**Authors:** Zhongmei Yang, Meng Wang, Dean S. Venables, Jun Chen

**Affiliations:** 1 China Machinery Industry Federation Key Laboratory of Multiphase Flow Measurement, School of Energy and Power Engineering, University of Shanghai for Science and Technology, Shanghai 200093, China; 2 Department of Chemistry and Sustainability Institute, University College Cork, Cork, Ireland; d.venables@ucc.ie

**Keywords:** SVOCs, absorption cross sections, integrated absorption, 2-nitrophenol, naphthalene

## Abstract

This study presents a generalized, high-precision measurement system based on Integrated Absorption Spectroscopy (IAS) for determining gas-phase absorption cross sections of low-volatility organic compounds (LVOCs), particularly semi-volatile organic compounds (SVOCs) in the atmosphere. Accurate cross sections and their temperature dependence are essential for modeling atmospheric and high-temperature processes. We coupled a temperature-controlled inlet and cell (473 K) with a nitrogen carrier gas to measure the cross sections of 2-nitrophenol (2-NP) and naphthalene from 250 to 400 nm. At 473 K, peak cross sections for 2-NP were 2.31 × 10^−17^ cm^2^/molecule at 260 nm and 1.16 × 10^−17^ cm^2^/molecule at 335 nm. For naphthalene, values between 258 and 280 nm decreased from 1.62 × 10^−17^ to 1.28 × 10^−17^ cm^2^/molecule. Thermally induced spectral broadening and reduced peak cross sections align with thermodynamic theory. These high-temperature data resolve discrepancies among low-temperature datasets. For example, our maximum cross section for 2-NP (300–400 nm) is 29% lower than that reported by Chen et al. (293 K), whereas the value from Sangwan and Zhu (295 K) is 86.8% lower than Chen’s, supporting the higher reliability of Chen’s data. The IAS method thus offers a robust approach for quantifying absorption cross sections under atmospherically relevant conditions.

## 1. Introduction

The accurate determination of gas-phase absorption cross sections for low-volatility organic compounds (LVOCs) remains a significant challenge in atmospheric chemistry research. Atmospheric organic compounds are classified by saturation concentration (C), with LVOCs (C < 10^6^ μg/m^3^) comprising a broad category that includes the critical subclass of semi-volatile organic compounds (SVOCs, C between 10^−1^ and 10^4^ μg/m^3^), which are distinguished by their significant gas–particle partitioning behavior [1]. SVOCs, an important subset of LVOCs, are particularly important due to their key roles in atmospheric processes. As key components of atmospheric organic aerosols, SVOCs primarily originate from direct emission sources such as vehicular exhaust, biomass burning, and coal combustion [1,2,3,4,5]. These compounds not only pose direct threats to human health [6,7] but also profoundly influence atmospheric oxidation capacity [8], secondary organic aerosol (SOA) formation, and climate effects [9,10,11].

The temperature of 473 K (~200 °C) was selected to simulate conditions in near-source combustion plumes and wildfire emissions, where temperatures can exceed 400 K. This elevated temperature helps prevent condensation of low-volatility species and provides absorption cross sections relevant to high-temperature atmospheric processes. This approach aligns with recent advancements in high-temperature laser spectroscopy, where elevated temperatures have been shown to significantly suppress molecular adsorption on chamber walls, thereby improving measurement accuracy [12,13].

Among SVOCs, nitrophenols and polycyclic aromatic hydrocarbons (PAHs) are particularly noteworthy due to their photochemical activity. Nitrophenols participate in ozone generation, OH radical cycling, and nitrogen oxide transformations [14,15,16]. As the smallest and most well-studied polycyclic aromatic hydrocarbon (PAH), naphthalene presents specific experimental challenges due to its low vapor pressure, which contributes to significant discrepancies in reported absorption cross sections. The atmospheric fate of these compounds, such as 2-nitrophenol (2-NP) and naphthalene, is governed by their gas-phase absorption characteristics in the near-ultraviolet range (250–400 nm), which directly determine their photolysis rates. Consequently, precise absorption cross-section measurements are critical for modeling atmospheric photochemistry.

Research on these molecules faces several key challenges: First, experimental data on gas-phase absorption cross sections for 2-NP and naphthalene at elevated temperatures (>400 K)—conditions primarily relevant to near-source combustion plumes and wildfire emissions—remain scarce. Second, discrepancies exceeding 45% exist in the reported absorption cross sections of naphthalene within the 250–290 nm range in spectral databases, introducing substantial uncertainties into atmospheric chemistry models. These discrepancies are largely due to experimental difficulties in measuring low-vapor-pressure species under combustion-relevant conditions. Third, conventional measurement techniques exhibit significant limitations: differential optical absorption spectroscopy (DOAS), when used for spectroscopic measurement of these species in the atmosphere, is constrained by optical path design in capturing broad absorption features [17,18]; cavity ring-down spectroscopy (CRDS) is challenged by significant wall losses that compromise gas-phase stability of low-volatility species [19,20]; solution-phase measurements [21] suffer from solvent-induced red-shifting of absorption peaks; while offline analytical methods are hampered by complex pretreatment procedures, low temporal resolution [22,23], and sampling losses.

This study is based on a high-precision measurement system of integrated absorption principles [24] for the quantitative determination of gas-phase absorption cross sections of LVOCs. We focus on SVOCs as representative LVOCs of interest in the Earth’s atmosphere, and potentially in astronomy. Using 2-NP and naphthalene as representative analytes, the setup enables direct measurement of gas-phase molecules. The research aims to deliver high-confidence quantitative absorption cross-section data for SVOCs, establish a robust methodological foundation for accurate gas-phase measurement while avoiding the limitations of solvent red shift and wall adsorption losses.

## 2. Results and Discussion

### 2.1. Absorption Cross Section of Naphthalene

**Figure 1:**
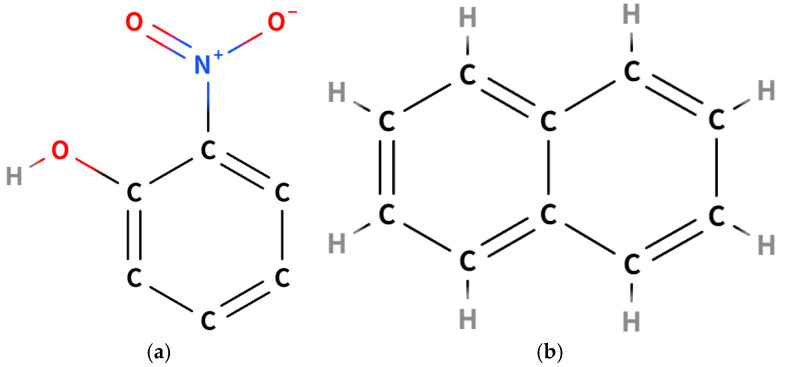
Schematic diagram of the molecular structures: (**a**) 2- nitrophenol; (**b**) naphthalene.

The molecular structures of the two key compounds investigated in this study, 2-NP and naphthalene, are depicted in Figure 1. As the simplest PAH, naphthalene possesses a rigid planar structure and an extended π-conjugated system. Its planar conjugated framework consists of two fused coplanar benzene rings, where carbon atoms adopt sp^2^ hybridization to form a σ-skeleton, while ten perpendicular p-orbitals constitute a delocalized π-system across ten atomic centers, resulting in partial bond length equalization. This delocalized π-system exhibits a HOMO–LUMO energy gap of 4.74 eV [25], leading to characteristic absorption in the 250–290 nm UV region. Schematic representations of its molecular structure and UV absorption electronic transitions are provided in Figure 1b and Table 1.

As presented in Table 1, the 220–230 nm region exhibits strong absorption, the 230–290 nm range features a multi-peak structure with pronounced vibrational fine features [25,26,27,28].

**Figure 2:**
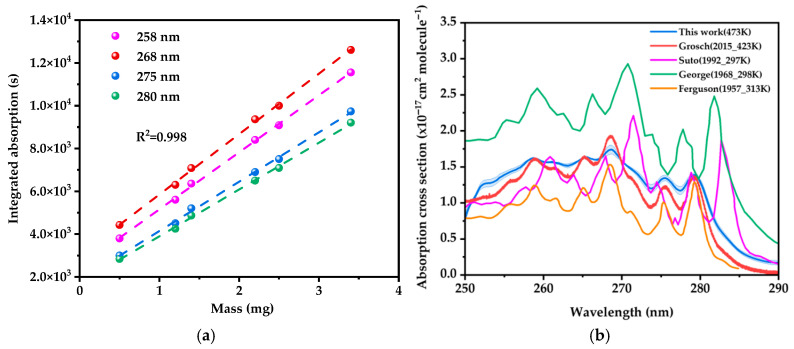
(**a**) Integrated absorption at four wavelengths with increasing mass of naphthalene. (**b**) Absorption cross section of naphthalene in the 250–290 nm wavelength range and reported literature spectra.

After integrating the area under the peak of the optical thickness (ln(*I*_0_/*I*)), the calculated absorption cross sections are shown in Figure 2b. The integrated absorption varied linearly with the amount of naphthalene in the cell (Figure 2a). Figure 2b presents the absorption cross section of gas-phase naphthalene in the 250–290 nm wavelength range, with 1σ uncertainty intervals.

Figure 2a displays the variation in absorbance versus mass (0.5–3.4 mg) for naphthalene vapor at four characteristic UV wavelengths: 258, 268, 275, and 280 nm. Significant linear dependence (*R*^2^ > 0.99) is observed at all wavelengths, consistent with the fundamental prediction of the Lambert–Beer law. The monotonically decreasing slope with increasing wavelength aligns with the distinctive absorption cross section profile of naphthalene in the UV region.

As shown in Figure 2b, the absorption cross section of gas-phase naphthalene near 275 nm measured in this work (1.35 × 10^−17^ cm^2^/molecule at 473 K) agrees well with the temperature-dependent trend reported by Grosch [27] et al. (1.23 × 10^−17^ cm^2^/molecule at 423 K). Both datasets demonstrate characteristic thermally induced band broadening, erosion of vibronic fine structures, and a red shift in the absorption maximum—consistent with a reduction in peak cross section at elevated temperatures. To further evaluate the oscillator strength independent of temperature-induced spectral shifts, the integrated absorption cross sections over the 250–290 nm range were compared across studies. The integrated value obtained in this work is (4.74 ± 0.20) × 10^−16^ cm^2^·nm/molecule (mean ± standard deviation), demonstrating excellent experimental precision. This value shows remarkable agreement with those derived from Grosch et al. (4.26 × 10^−16^ cm^2^·nm/molecule) and Suto [26] et al. (4.44 × 10^−16^ cm^2^·nm/molecule), confirming conservation of oscillator strength across a wide temperature range (297–473 K) and validating the absolute calibration methods employed in these studies.

Notable discrepancies emerge when comparing with previously published datasets: Suto et al. (1.42 × 10^−17^ cm^2^/molecule at 297 K) reported high-resolution spectra revealing vibrational fine structures, though absolute cross sections may be influenced by calibration reference selection; George [29] et al. (2.02 × 10^−17^ cm^2^/molecule at 298 K) exhibited higher values likely originating from solvent-phase extrapolation, introducing bathochromic shifts and intensified oscillator strengths. The significantly larger integrated value from George et al. (7.45 × 10^−16^ cm^2^·nm/molecule) further supports this overestimation due to solvent-phase extrapolation artifacts, far exceeding the uncertainty range of our measurements. Ferguson et al. [30] (≈ 1.01 × 10^−17^ cm^2^/molecule at 313 K) showed systematic underestimation, attributable to collisional broadening under argon atmosphere and reliance on obsolete vapor-pressure models. This interpretation is reinforced by their lower integrated cross section (2.99 × 10^−16^ cm^2^·nm/molecule), which falls outside the uncertainty range of our integrated value and aligns with the effects of collisional broadening and outdated vapor-pressure models.

The variations in reported cross sections and spectral resolution are predominantly attributable to temperature effects and methodological differences in concentration calibration. Low-temperature measurements (≤313 K) preserve sharp vibrational features due to reduced Doppler broadening and minimal thermal population of excited vibrational states. In contrast, high-temperature results (≥423 K) exhibit extensive band broadening and reduced peak intensities—consistent with elevated rovibrational excitation, increased collision rates, and thermal occupation of hot bands—while approximately conserving the integrated absorption intensity. These observations align with temperature-dependent line-shape theories and vibrational channel-opening mechanisms.

These discrepancies in absorption cross sections can lead to significant uncertainties in modeled photolysis rates. For example, an overestimation of cross sections may result in underestimated atmospheric lifetimes, affecting predictions of SOA formation and oxidative capacity in plumes. The high-temperature data provided in this study helps resolve these uncertainties by establishing reliable reference values under conditions relevant to near-source emissions.

Together with the work of Grosch et al., this study quantitatively establishes the thermal attenuation behavior of naphthalene UV absorption. The integrated cross section comparison, supported by the high precision of our measurements, effectively resolves prior literature discrepancies and establishes a consistent benchmark for the naphthalene UV band oscillator strength. Conversely, the high-resolution, low-temperature data from Suto et al. provide critical benchmarks for vibronic assignment and intrinsic spectroscopic characterization.

### 2.2. Absorption Cross Section of 2-NP

2-NP is an important atmospheric pollutant featuring ortho-substituted nitro (–NO_2_) and hydroxy (–OH) groups on its benzene ring (Figure 1a). The nitro group, as a strong electron-withdrawing moiety, forms an intramolecular charge transfer (ICT) system with the electron-donating hydroxy group, profoundly modulating its electronic excitation behavior. The two characteristic UV absorption bands of 2-NP are assigned to distinct electronic state transitions and are summarized in Table 2 [31,32,33].

**Figure 3:**
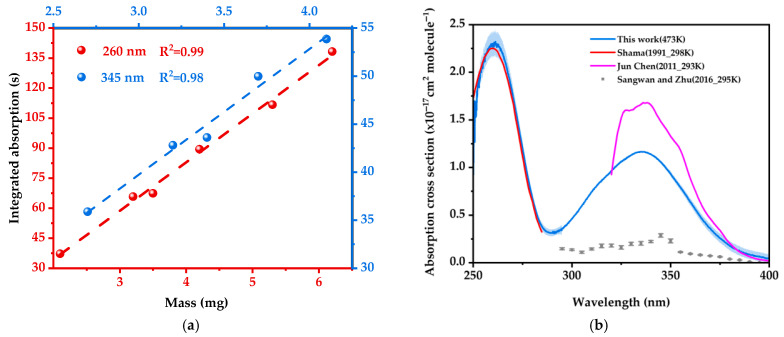
(**a**) Integrated absorption at two wavelengths with increasing mass of 2-NP. (**b**) Absorption cross section spectrum of 2-NP in the 250–400 nm wavelength ranges from this work and from previous reports in the literature.

Integration of the peak area over time yielded absorption cross sections in both spectral bands. These cross sections were then concatenated to obtain the gas-phase 2-NP absorption cross section spectrum spanning 250–400 nm, with 1σ uncertainty intervals as depicted in Figure 3b. As illustrated in Figure 3a, the integrated absorption varied linearly with the amount of 2-NP in the cell.

The sharp absorption peak of 2-NP at 260 nm originates from a localized π→π* transition of the benzene ring (*S*_4_, HOMO-1 → LUMO). Its slight red shift (compared to intrinsic benzene absorption at ~254 nm) is driven by the electron-withdrawing conjugation of the ortho-nitro group, while steric hindrance significantly suppresses nitro-benzene coplanarity (dihedral angle > 30°). This restriction on conjugation extension results in a steep potential energy surface (reorganization energy λ ≈ 0.3 eV) and high oscillator strength (*f* = 0.19), collectively yielding the peak’s sharp, temperature-insensitive characteristics reflective of a rigid molecular framework.

Conversely, the absorption band of 2-NP centered at 335 nm is attributed to the S_0_ → S_1_ transition, which is a symmetry-allowed π→π* excitation with pronounced intramolecular charge transfer (ICT) character. This ICT nature is facilitated by the ortho-substituted –NO_2_ and –OH groups, which form an electron push–pull system via a strong intramolecular O–H⋯O hydrogen bond (~1.8 Å). At elevated temperatures (473 K), thermal weakening of the hydrogen bond leads to broadening and a red shift in the absorption band. Furthermore, the spectral shape changes can be rationalized by the temperature-dependent behavior of excited-state intramolecular proton transfer (ESIPT) pathways, as reported in the literature [34]. In this model, excitation into the low-energy edge of the absorption band (populating the S_1_ state) results in a higher ESIPT yield (~70%), whereas higher-energy excitation (into S_3_/S_4_) shows reduced proton transfer efficiency (~27%) [34]. This differential reactivity offers a compelling interpretation for the specific diminishment of absorption above 300 nm observed under our high-temperature conditions.

From Figure 3b, the absorption cross section of gas-phase 2-NP determined here aligns closely with that reported by Shama et al. [35]. For the π to π* band peaking at 260 nm, the strong agreement in the spectral overlap region (280–330 nm) further supports the accuracy of our results. Our maximum is 29% lower than that reported by Chen [36] et al. The absorption peaks reported by Chen et al. for 2-NP are comparable in position to those observed in this study but also exhibit small spectral structures on top of the main absorption band. This discrepancy can be primarily attributed to temperature effects: the elevated temperature (473 K) used in our measurements leads to spectral broadening and a reduced maximum absorption cross section—consistent with typical thermodynamically expected behavior and similarly observed in our naphthalene reference measurements. Chen et al. measured the absorption cross section using incoherent broadband cavity-enhanced absorption spectroscopy (IBBCEAS) in an atmospheric simulation chamber. Their system employed high-reflectivity mirrors (R > 99.6%) across the 300–450 nm range to form the optical cavity. Although unaccounted slowly varying mirror reflectivity features could potentially introduce spectral artifacts, the overall differences are more consistently explained by temperature-dependent spectral shape changes.

Sangwan and Zhu [20] measured the gas-phase absorption cross sections of 2-NP at discrete single wavelengths under low-pressure conditions using cavity ring-down spectroscopy (CRDS). Their reported values were much lower than those of Chen et al. In this work, the gas-phase 2-NP concentration was calculated based on the intracavity pressure, making absolute pressure measurement critical for accurate concentration quantification. Furthermore, potential biases such as molecular losses to chamber walls and the deposition of sample coatings on optical cavity mirror surfaces may also introduce uncertainties in the measured absorption cross sections.

The quantification of mirror reflectivity can also affect the absorption cross section measurements. The absorption cross section of gas-phase 2-NP reported in this study was measured at a temperature of 473 K, and typically, an increase in temperature results in a decrease in the gas absorption cross section [37,38]. This is what we observe with the spectrum from Chen and co-workers, whereas the Sangwan and Zhu spectrum is inexplicably lower. Owing to the general agreement in the shape and magnitude of the spectrum measured with a substantially different measurement principle, we conclude that the room-temperature cross section reported by Chen is more reliable than that of Sangwan and Zhu.

## 3. Materials and Methods

### 3.1. Materials

Naphthalene (analytical standard grade, GC) and 2-NP (analytical standard grade, GC) were sourced from Shanghai Aladdin Biochemical Technology Co., Ltd. (Shanghai, China).

### 3.2. Experimental Setup

A spectroscopic system for measuring absorption cross sections at elevated temperatures based on the principle of integrated absorption was constructed in the laboratory. This system consisted of a light source, a detection cell, and a fiber spectrometer.

**Figure 4:**
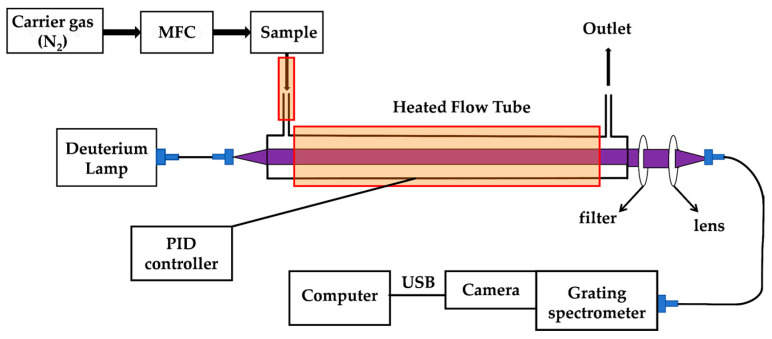
Schematic diagram of the flow absorption cross section measurement system.

The schematic diagram of this apparatus is presented in Figure 4, featuring temperature-controlled heating units at both the gas inlet and detection cell to prevent gas condensation in the sample transfer line and optical chamber. Key components of the spectrometer are cataloged in Table 3.

### 3.3. Integrated Absorption Measurement Principle

The fundamental principle of integrated absorption is also based on the Lambert–Beer law. It involves integrating the time-dependent absorption of a target gas flowing through the optical path over a defined time period, thereby obtaining the cumulative absorption of the gas during that interval [24]:

(1) $$\ln\left(\frac{I_{0}\left(t\right)}{I\left(t\right)}\right)=N\left(t\right)\cdot \sigma \cdot d=\frac{n\left(t\right)}{V_{C}}\cdot \sigma \cdot d$$

where *I*_0_(*t*) and *I*(*t*) denote the transmitted light intensities in the absence and presence of the absorbing gas within the detection cell, respectively; *N*(*t*) represents the time-averaged molecular number density; *n*(*t*) is the total number of target gas molecules in the detection cell; *V*_C_ is the volume of the detection cell; *σ* signifies the absorption cross section; and *d* corresponds to the length of the detection cell. Integrating the logarithm of the transmitted light intensity ratio (termed the optical depth) over time, we obtain the integrated absorption *A*, whose variation per molecule is expressed as:

(2) $$A=\int_{t=0}^{\infty }\ln\frac{I_{0}(t)}{I(t)}dt=\frac{\sigma d\tau }{V_{C}}$$

here *τ* denotes the residence time of a single molecule within the detection cell. For the integrated absorption contributed by the total number of gas molecules, *n_T_*:

(3) $$A_{T}=\frac{n_{T}\sigma d\tau }{V_{C}}$$

The integrated absorption *A_T_* in Equation (3) can be alternatively described as the total absorption contributed by a molecular ensemble with a constant number density *N*_C_ over a defined time interval:

(4) $$A_{T}=\ln\frac{I_{0}(t)}{I(t)}\tau =N_{C}\cdot \sigma \cdot d\cdot \tau$$

For a flowing gas stream, the number density is defined as the ratio of the total number of gas molecules to the total volumetric flow of gas:

(5) $$N_{C}=\frac{n_{T}}{V_{S}}$$

here *V*_S_ represents the total volume of the gas stream carrying analyte molecules. This can be expressed as the product of the volumetric flow rate *f* and time *t* ($V_{S}=f\tau$). Consequently, the integrated absorption *A_T_* is given by:

(6) $$A_{T}=\int_{t=0}^{\infty }\ln\frac{I_{0}\left(t\right)}{I\left(t\right)}dt=\left(\frac{n_{T}}{f}\right)d\sigma$$

Once the total number of gas molecules *n_T_*, volumetric flow rate *f*, and sample path length *d* in the detection cell are determined, the absorption cross section of the target gas can be derived through integration of the optical depth over the measurement period:

(7) $$\sigma =\frac{A_{T}f}{n_{T}d}$$

This result is experimentally convenient because the total number of molecules flowing through the cell is readily and accurately known from the sample mass or volume. This approach avoids the need for precise control of the target gas concentration, which can be challenging for low-volatility species.

### 3.4. Methods

The temperature of 473 K was optimized through a series of pre-experiments to ensure complete volatilization of 2-NP and naphthalene while avoiding thermal decomposition. This temperature also aligns with conditions reported in previous high-temperature spectroscopic studies, where temperatures above 415 K have been shown to significantly suppress molecular adsorption on chamber walls [12].

Prior to experiments, the detection cell was preheated to 473 K and purged with high-purity nitrogen (99.999%) for 30 min until it reached thermal stability. Precisely weighed solid samples were vaporized in a gas-washing bottle through controlled heating using a laboratory heat gun and transported into the detection cell using a nitrogen carrier gas. Spectral measurements for 2-NP were performed using a spectrograph with camera, covering two overlapping spectral regions to optimize signal quality: 280–409 nm with a 450 nm short-pass filter, and 200–330 nm with a 300 nm short-pass filter after grating adjustment. The 300 nm and 450 nm short-pass filters were selected to isolate specific spectral regions and minimize stray light. The 300 nm filter was used for the 200–330 nm range to enhance signal-to-noise in the deep-UV, while the 450 nm filter allowed measurement of the 280–409 nm range with reduced visible background. Excellent agreement was observed in the overlapping region (280–330 nm), confirming the consistency and reliability of the spectral data (Figure 3b).

**Figure 5:**
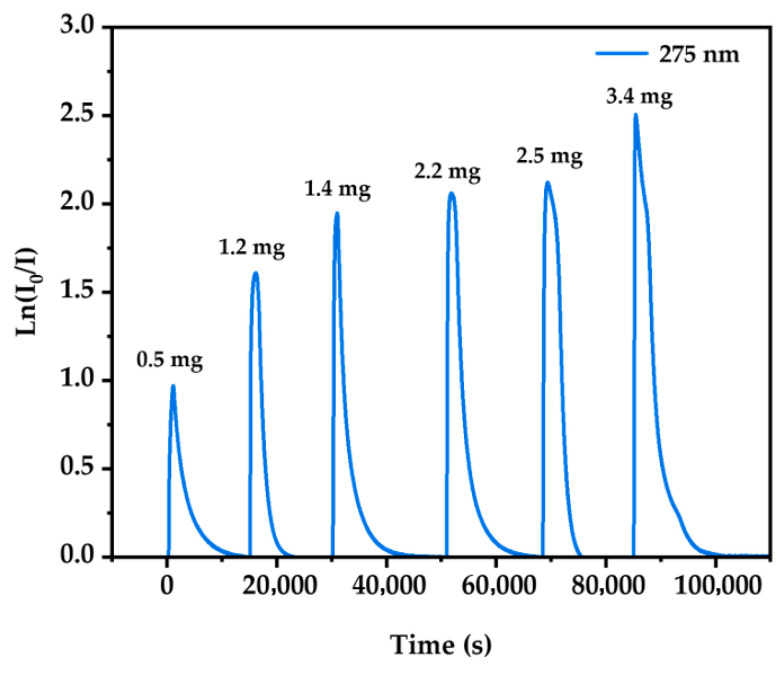
Time dependence of the optical depth at 275 nm for different masses of naphthalene to the gas cell.

For naphthalene, measurements were carried out using a fiber spectrometer across the 200–320 nm range, employing a 300 nm short-pass filter to minimize stray light. Multiple independent additions were conducted during each phase to ensure data reproducibility. Data below 250 nm were excluded due to the spectrometer’s low photon efficiency in this region, resulting in a poor signal-to-noise ratio (SNR < 3). For 2-NP, sample masses ranged from approximately 2.1 to 6.5 mg per weighing, while for naphthalene, masses between 0.5 and 3.4 mg were used. Six independent measurements were performed for each compound. The 1σ uncertainty is attributed to multiple sources, including instrument calibration, sample mass weighing (±0.1 mg), flow rate fluctuations (<±2%), and spectral reproducibility across repeated measurements (as shown by the error bars in Figure 2b and Figure 3b). Figure 5 illustrated the temporal profiles of the optical depth (ln(*I*_0_/*I*)) for naphthalene at 275 nm.

## 4. Conclusions

This study establishes an Integrated Absorption Spectroscopy platform that integrates thermodynamic control with spectral measurements to quantify gas-phase absorption cross sections of SVOCs. Regulation of the detection cell temperature at 473 K significantly suppressed wall adsorption, thereby enabling effective measurement of these difficult-to-handle, low-vapor-pressure species. For 2-NP, measured absorption cross sections in the 250–280 nm band aligned with literature values, while deviations at 300–400 nm can be primarily attributed to excitation-wavelength-dependent ESIPT dynamics. In contrast, the absorption cross sections of naphthalene in the 250–290 nm range showed excellent agreement with the high-temperature values reported by Grosch et al. (423 K). These results validate the IAS platform as a reliable quantitative tool for SVOCs, while providing essential spectral parameters for atmospheric research applications. Moreover, this approach can be extended to characterize larger polycyclic aromatic hydrocarbons (PAHs), which absorb in the near-UV region and are increasingly relevant in atmospheric chemistry and astrophysics. High-quality experimental cross sections of such compounds may also serve as critical benchmarks for evaluating computational chemistry predictions.

## Figures and Tables

**Table 1:** UV absorption transition characteristics of naphthalene.

Wavelength Range (nm)	Transition Energy Levels	Electronic Transition Type	Oscillator Strength (f)
220–230	S_0_ → S_3_	π → π*	0.059
230–290	S_0_ → S_2_ (^1^B_2u_ ← ^1^Ag)	π → π*	0.089

**Table 2:** UV absorption transition characteristics of 2-NP.

Wavelength Range (nm)	Transition Energy Levels	Electronic Transition Type	Oscillator Strength (*f*)
255–275	S_0_ → S_4_	π → π*	0.19
320–350	S_0_ → S_1_	π → π*	0.06

**Table 3:** Spectrometer components and specifications.

Module	Manufacturer	Model/Specifications	Key Features
Light Source System			
Deuterium lamp	Avantes (Apeldoor, The Netherlands)	Avalight-D-S-DUV	Spectral range: 175–400 nm
Emission fiber	Avantes (Apeldoorn, The Netherlands)	FC-UVIR400-0.5-ME	Core diameter: 400 μm
Reflective collimator	Thorlabs (Newton, NJ, USA)	RC04SMA-P01	2D-adjustable design
Absorption Cell and Spectral Detection System			
Quartz absorption cell	–	Custom-built	Length: 420 mm, Gas port ID: 8 mm
Precision balance	Ohaus (Parsippany, NJ, USA)	PWS224ZH	±0.1 mg
Temperature control unit	YuDian (Shanghai, China)	AI-206/207	Proportional–integral–derivative (PID)-controlled
Short-pass filters	Semrock (Rochester, NY, USA)	FF01-300/SP-25 (300 nm)	UV band cutoff
	Asahi Spectra (Torrance, CA, USA)	XHS0450 (450 nm)	Visible band cutoff
Focusing lens	–	*f* = 50 mm	Fiber optic coupling
Spectrograph	Andor Tech (Belfast, UK)	Kymera-328i-A	UV–Vis spectral analysis
CCD detector	Andor Tech (Belfast, UK)	DU420A-BU	High-sensitivity detection
Fiber spectrometer	Avantes (Apeldoorn, The Netherlands)	ULS2048XL-RS-EVO	Grating: 2400 lines/mm, slit50, 0.26–0.34, 200 nm–320 nm

## Data Availability

The data presented in this study are available upon request from the corresponding author.
